# A BIBLIOMETRIC ANALYSIS WITH DATA OPENALEX AND MINING METHODS OF 41 525 ABSTRACTS OF PAPERS ON THE HEALTH IMPACT OF AIR POLLUTION PUBLISHED BETWEEN 1960 AND 2022

**DOI:** 10.13075/ijomeh.1896.02537

**Published:** 2025

**Authors:** Bogdan Bochenek, Mateusz Jankowski, Joanna Wieczorek, Marta Gruszczyńska, Jarosław Pinkas, Mariusz Figurski

**Affiliations:** 1 Institute of Meteorology and Water Management – National Research Institute, Centre of Numerical Weather Prediction, Warsaw, Poland; 2 Centre of Postgraduate Medical Education, School of Public Health, Warsaw, Poland

**Keywords:** review, PM_2.5_, air pollution, trends, health, bibliometric analysis

## Abstract

Exposure to air pollution is a significant risk factor for non-communicable diseases. This bibliometric analysis with data mining methods aimed to identify the most common air pollutants and health effects mentioned in research on the health effects of air pollution published in 1960–2022. The OpenAlex database and OpenAlexR package were used to retrieve abstracts of scientific papers on the health impact of air pollution published in 1960–2022. Publication year, type of air pollutant, type of diseases analyzed in the study, and affiliation of the authors were analyzed using data mining methods. Out of 41 525 papers published in 1960–2022, 22.3% (N = 9255) listed particulate matter 2.5 (PM_2.5_) in the abstract at least once, 13.2% (N = 55 011) listed PM_10_, 11.6% listed carbon monoxide (CO) (N = 4829), 11.5% (N = 4784) listed nitrogen dioxide (NO_2_), 7.5% listed sulfur dioxide (SO_2_) (N = 3106), and 7.1% of papers listed ozone (O_3_) (N = 2943). Respiratory diseases were the most common health effects. Most of the papers (N = 1880) were focused on PM_2.5_. The most common co-occurrence patterns included the impact of PM_2.5_ on lung, heart, and asthma. In total, in 1960–2022 authors from 165 different countries published at least 1 paper on the health effects of air pollution. This study provided bibliometric data on the number and topics of papers on the health impact of air pollution published in the past 60 years. Most of the papers were published by authors from the global North with a very limited number of papers on air pollution and health published by the authors from Africa and South America.

## Highlights

- In this bibliometric analysis OpenAlex database and OpenAlexR package were used.
- A total of 41 525 abstracts of scientific papers were included in this analysis.
- Most of the research are focused on particulate matter 2.5 (PM_2.5_) and respiratory health.
- There was a limited number of authors from Africa and South America.
- This study provides full overview of research on the health impact of air pollution.

## INTRODUCTION

Exposure to air pollution is a significant risk factor for non-communicable diseases, accounting globally for 7 million premature deaths every year [1,2]. According to the World Health Organization (WHO), 99% of the global population lives in places where air pollution levels put them at increased risk for diseases [1]. Particulate matter (PM), carbon monoxide (CO), ozone (O_3_), nitrogen dioxide (NO_2_), and sulfur dioxide (SO_2_) are so far well-known air pollutants of a major public health concern [1,3,4]. Ambient (outdoor) air pollution is mostly caused by household heating systems, burning fossil fuels, motor vehicles and transportation, industrial facilities, and forest fires [1,5]. While household (indoor) air pollution is mainly dedicated to incomplete combustion of solid fuels for cooking or heating [1,6].

Exposure to air pollution may cause both acute and chronic health effects [5,7]. Long-term exposure to air pollution is linked with an increased risk of respiratory diseases, cardiovascular diseases, allergic rhinitis, and lung cancer. Acute exposure to air pollution may evoke exacerbation of respiratory diseases like asthma or chronic obstructive pulmonary diseases [5,7,8].

Ambient air pollution is a significant environmental health problem affecting low-, middle as well as high-income countries [1,9,10]. Exposure to air pollution is observed both in rural and urban areas [1,10]. The highest concentration of air pollution is usually observed in urban centers, industrial regions, and transportation corridors [10].

World Health Organization published the first Global Air Quality Guidelines in 1987 [11]. Since then, guidelines have been updated several times [11,12]. These guidelines provided recommendations on air quality levels and interim targets for 6 key air pollutants [11]. Compared to the first editions of the WHO Global Air Quality Guidelines, the latest guidelines from 2021 provided a much stronger body of evidence to show how air pollution affects human health at even lower concentrations than previously understood [11,12]. Due to the high health burden of air pollution, the WHO called member states to implement mitigation strategies, monitor air pollution, and improve air quality [13]. Emission control policies, renewable energy adoption, public transportation enhancement, green urban planning, and reforestation are effective public policies that may limit the emission of air pollutants [14]. The level of implementation of air pollution mitigation strategies differs across the countries, wherein there is a lag in the implementation of air-quality policies in low- and middle-income countries [15].

Scientific data, both from the regional, national, and multi-center studies may support the implementation of effective mitigation strategies, tailored to the local environments [16,17]. However, environmental health research is less developed compared to clinical medicine research.

Artificial intelligence (AI) and AI-related analytical methods like data mining and machine learning are rapidly developing and spreading in many areas, supporting the growth in publication numbers and the emergence of further complex interdisciplinary analysis. The development of digital tools poses a new opportunity for scientific analysis, including big data analysis. Bibliometric analysis (BA) is a common method for exploring and analyzing large volumes of scientific data. It is useful to get a reliable, simplified and structured, and specified quantitative estimate of the rise in scientific publications, especially in the medical field [18]. Bibliometric analysis may also help researchers to track the status, development and evolution, identify gaps and categorize knowledge in a field using traditional literature review methods, like, e.g., narrative and meta-analysis [19]. Bibliometric analysis in the broad issue of the impact of air pollution on health, defendant to take a holistic view and may summarize trends in air pollution research and the research topics of high interest to scientists [20]. Moreover, global analysis of data on the health impact of air pollution may indicate countries and regions that are leaders in environmental health research on the health impact of air pollution [16,20,21]. While bibliometric analyses of the impact of air pollution on individual diseases or with consideration of vulnerable groups are already available in the medical sciences, a broader and global view has so far been lacking. And such a one could lead to better identification of potential areas of research, and could also encourage exploration of the topic by research teams from related fields as well.

This bibliometric analysis aimed to characterize the most common global scale air pollutants and health effects mentioned in research on the health effects of air pollution published in 1960–2022 using data mining methods and a new bibliographic catalogue OpenAlex.

## METHODS

### Study design

This study is BA of scientific papers on the health impact of air pollution using data mining methods. OpenAlex database and OpenAlexR package were used to retrieve abstracts of scientific papers on the health impact of air pollution published in 1960–2022 [22,23]. OpenAlex is a non-profit, open catalog of the global research system, started operating in January 2022 [22,24,25] and is rapidly gaining popularity. Especially, as it combines access to records from databases such as PubMed, Web of Science, Scopus, Cinahl, and the Cochrane Library databases, while allowing the exclusion of repetitive records from the analysis.

Databases like Scopus and Web of Science considered individually, which have so far provided the basis for research, while, as the scientific community raises, they under-represent some disciplines and regions of the world [24,25]. The BA is typically based on text analytics, which means that keywords must be defined before the search can begin. A set of globally most typical pollutants was generated, as well as, based on WHO reports and previous reviews [7], a set of health outcomes organized into categories, which are described in the following part of this chapter. It is worth noting that using this approach is not possible to prepare category “other” diseases, as only precise keywords could be used.

Papers (abstracts published in English) on the health impact of air pollution were identified and indexing using the following keywords: „air pollution” and „health”. At the next stage, the classification of works considered for further analysis was done by searching for established concepts. In OpenAlex database each work is tagged with multiple concepts, based on the title, abstract, and the title of its host venue [24,25]. The tagging is done using an automated classifier that was trained on Microsoft Academic Graphs (MAG's) corpus. The authors implement the classifier using selected concepts as follows: “medicine,” “environmental health,” “disease,” “internal medicine,” “air pollution,” “public health.” As the effect of the abstract analysis (using the function of “abstract search mode” with the OpenAlex package), a dataset covering 41 525 unique abstracts was extracted. The dataset was downloaded and metadata including authors, title, affiliations, publication year, and abstracts were extracted [22]. This study included all types of air pollution research including toxicology, human laboratory studies, epidemiology, and exposure assessment studies. The absolute numbers of papers published in particular years were presented.

### Text analysis and identification of the most common phrases mentioned in the abstracts

The abstracts were first tokenized into individual words using the tidy text package [26]. This tokenization process dismantled sentences into words, which serves as the preliminary step for subsequent frequency analysis. Common stop words were excluded from the dataset to ensure that only meaningful, content-related words were included in the analysis. The frequency of each remaining term was computed, and the terms were then sorted based on their occurrence. Additionally, specific words and numerals that were deemed irrelevant or non-informative for the context of the research were manually filtered out of the dataset. The visualization was configured to display up to 100 words, ordering them by frequency and omitting words with a frequency below one. Moreover, text analysis was carried out to identify the number of abstracts that mentioned (at least once) the most important air pollutants.

A database of abstracts was compiled into a corpus using the tm package. The text was standardized through several preprocessing steps, including conversion to lowercase, removal of punctuation, exclusion of standard English stop words, and stripping of additional whitespace to facilitate accurate text analysis. A curated list of relevant terms, representing diseases and pollutants of interest (e.g., “cancer,” “respiratory,” “pm10,” “no2”), was utilized to filter the corpus. Major air pollutants of public health concern were identified following the WHO publications and included: PM_2.5_, PM_10_, SO_2_, NO_2_, O_3_, and CO [1,3,4]. Major groups of diseases were identified based on the most common groups of health conditions linked to air pollution exposure: respiratory, cardiovascular, cancer, immune, mental, and birth conditions [5,7,8]. Due to the technical reasons and possibilities of research tools used in this study, analyses were focused on major air pollutants and groups of health effects. Text tokenization was executed to deconstruct the abstracts into words, after which only the terms from the predefined lists – which were highlighted in previous systematic reviews, e.g., in the study by Dominski et al. [27], were retained. Each abstract was processed to ensure that terms representing diseases and pollutants were only counted once, regardless of their frequency within an individual abstract. This methodological choice emphasizes the presence rather than the prevalence of terms in each document. A co-occurrence matrix was constructed to quantify the instances where disease and pollutant terms appeared within the same abstracts, thereby suggesting potential associations between them for further exploration.

### Publication year

For each publication in the database, the parameter “publication_year” was extracted, and summed for each year from 1960 to 2022.

### Geographical location of authors

Author affiliations were identified and geocoded using OpenAlex tools. For each publication in the database, the parameters “author” and “institution_country_code” were extracted and counted. In the case of multiple authors from one country in a publication, all authors are counted separately as individual counts from a given country in a final database. Therefore, the total count for each country is not equal to the number of individual authors, as 1 author that published several publications will be counted multiple times, but it is a number of authors affiliations from this country. Data were presented with a logarithmic scale.

## RESULTS

### Air pollution health-related topics in scientific papers

A total of 41 525 papers on the health impact of air pollution were analyzed. There was a strong research emphasis on the environmental determinants of health, particularly air quality and its effects on human health outcomes in papers on the health effects of air pollution. Out of 41 525 papers included in this bibliometric analysis, 22.3% (N = 9255) listed PM_2.5_ in the abstract at least once, 13.2% (N = 5011) listed PM_10_, 11.6% listed CO (N = 4829), 11.5% (N = 4784) listed NO_2_, 7.5% listed SO_2_ (N = 3106), and 7.1% of papers listed O_3_ (N = 2943).

### Changes in the annual number of published papers on the health impact of air pollution

**Figure 1.**
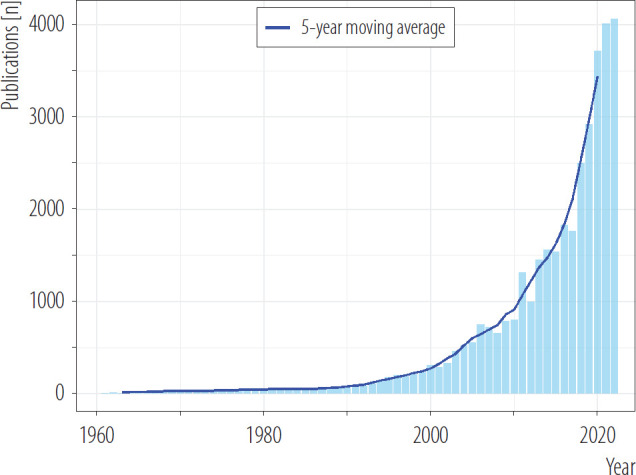
Annual number of scientific papers on the health impact of air pollution published published in 1960–2022 with a 5-year moving average (crude numbers), review conducted in 2024

**Figure 2.**
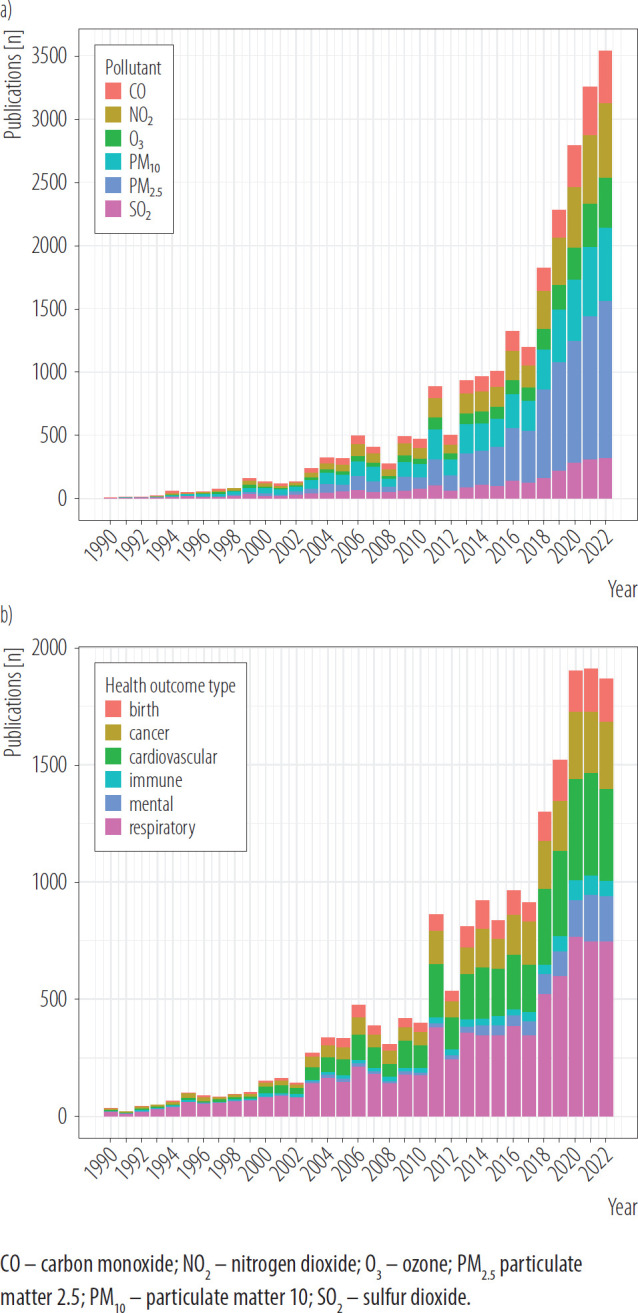
Annual number of papers concerned air pollutants and its health impact published in 1960–2022 specified by a) the pollutant, b) health outcome type in OpenAlex English language database, review conducted in 2024

Figure 1 presents the trends in the annual number of publications on the health effects of air pollution over time, alongside a 5-year moving average. A noticeable increase in the number of publications begins after 1990, marking the start of a period of growing research interest in this field. A particularly dynamic and sharp rise in publication volume is evident from the early 2000s (Figure 1). The most striking surge occurred after 2010, with the moving average displaying a steep upward trajectory, reflecting a significant expansion in published papers, with over 1000 publications per year since 2011. The highest number of publications was observed in 2022 when the annual number of published papers on the health effects of air pollution reached the peak of 4068 publications (accounting for almost 10% of all published papers). Between 1990 and 2022, differences in the number of publications by pollutant were observed (Figure 2a). Since 2010, air pollution research has been predominated by the research on particulate matter (Figure 2a). Between 1990 and 2022, a systematic decrease in the number of papers focused on gaseous pollutants like SO_2_ or CO was observed, with a dynamic increase in the number of papers on particulate matter (Figure 2a).

Between 1990 and 2022, differences in the most common health effects of exposure to air pollution analyzed in scientific papers were observed (Figure 2b). However, in all analyzed years, respiratory diseases were the most common health effects of air pollution analyzed in the scientific papers (Figure 2b). In the analyzed period, the number of scientific papers on the cardiovascular effects of exposure to air pollution stably increased. In total, 1360 abstracts mentioned “diabetes.” In the past 5 years, a dynamic increase in the number of papers on the impact of air pollution on mental health was observed (Figure 2b).

### Co-occurrence of air pollutants and health effects analyzed in scientific papers

**Figure 3.**
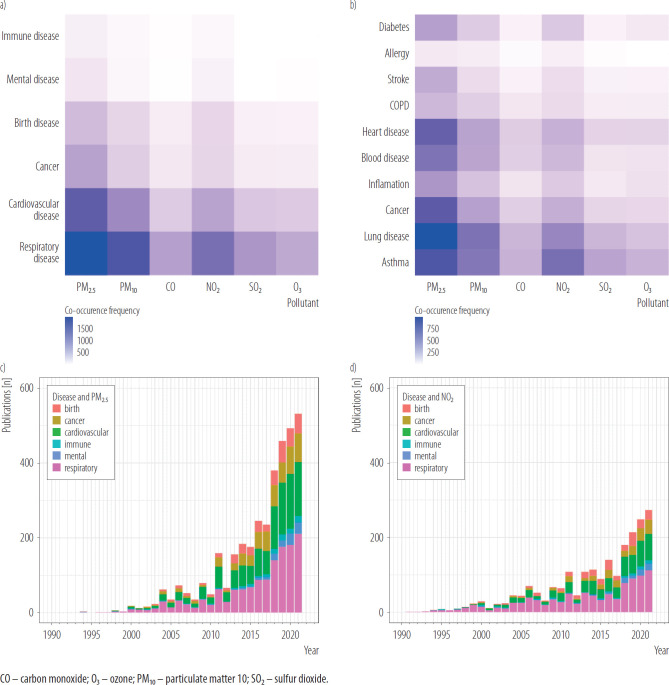
Co-occurrence of diseases and air pollutants in 41 525 English language scientific papers on the health impact of air pollution published in 1960–2022: a) by group of diseases, b) by particular diseases, c) by disease co-occurring with particulate matter 2.5 (PM_2.5_), d) by diseases co-occurring with nitrogen dioxide (NO_2_), review conducted in 2024

Most of the papers (N = 1880) published in 1960–2022 were focused on the impact of PM_2.5_ on the respiratory system (Figure 3a). In total, 1578 papers were focused on PM_10_ exposure and its impact on the respiratory tract. Moreover, 1417 papers analyzed the impact of PM_2.5_ on cardiovascular diseases and 925 papers analyzed the impact of PM_10_ on cardiovascular diseases. Among published papers, 1218 were focused on the impact of NO_2_ exposure on respiratory health, 704 analyzed the impact of NO_2_ on cardiovascular health, and approx. 300 papers were focused on the impact of NO_2_ exposure on cancer risk and birth outcomes (Figure 3a).

The co-occurrence of air pollutants and the 10 most common health conditions listed in scientific papers on the health impact of air pollution is presented in Figure 3b. Most of the papers were focused on the impact of PM_2.5_ exposure on lung, heart, and asthma. Moreover, the impact of PM_10_ exposure and NO_2_ on asthma, lung, cancer, blood, heart, and diabetes were also analyzed in several hundreds of papers (approx. 300–350 papers per disease).

Between 2018 and 2022, 929 papers on respiratory diseases co-occurring with PM_2.5_ exposure and 695 papers on cardiovascular diseases co-occurring with PM_2.5_ exposure have been published (Figure 3c). At the same time, 483 papers on respiratory diseases co-occurring with NO_2_ exposure and 303 papers on cardiovascular diseases co-occurring with NO_2_ exposure have been published (Figure 3d). Crude numbers are presented in Table 1 and 2.

**Table 1.** Annual number of papers on co-occurrence of diseases and air pollutants published in 1960–2022 (crude numbers), review conducted in 2024

Health effect	Papers by pollutant [n/year]					
	PM_2.5_	PM_10_	CO	NO_2_	SO_2_	O_3_
Respiratory	1880	1578	731	1218	827	653
Cardiovascular	1417	925	386	704	414	392
Cancer	717	371	192	314	167	159
Birth	499	317	157	303	131	126
Mental	206	93	47	118	50	56
Immune	134	85	53	84	43	41
Asthma	785	571	301	617	351	295
Lung	942	595	281	497	280	235
Cancer	717	371	192	314	167	159
Inflammation	421	235	116	207	103	126
Blood	589	350	192	273	115	122
Heart	691	358	197	303	174	177
Chronic obstructive pulmonary disease	270	189	110	152	88	86
Stroke	329	148	74	142	72	76
Allergy	103	85	53	83	42	32
Diabetes	375	201	73	215	75	98

CO – carbon monoxide; NO_2_ – nitrogen dioxide, O_3_ – ozone; PM_2.5_ – particulate matter 2.5; PM_10_ – particulate matter 10; SO_2_ – sulfur dioxide.

**Table 2.** Co-occurrence of diseases and selected pollutants in papers on co-occurrence of diseases and air pollutants published in 1960–2022 (crude numbers), review conducted in 2024

Co-occurrence	Papers [n]												
	2010	2011	2012	2013	2014	2015	2016	2017	2018	2019	2020	2021	2022
Disease and PM_2.5_													
birth	7	11	7	24	26	23	29	18	39	58	49	53	57
cancer	2	25	5	19	32	28	45	52	57	54	73	77	89
cardiovascular	16	57	25	47	55	48	73	62	113	138	147	144	153
immune	3	3	0	4	5	7	4	8	13	16	12	17	14
mental	0	0	0	1	4	2	7	7	18	17	31	30	28
respiratory	21	63	29	61	62	68	87	88	140	176	181	211	221
Disease and NO_2_													
birth	9	12	9	17	17	15	27	12	16	37	24	26	26
cancer	4	16	3	7	13	13	22	19	16	24	33	38	33
cardiovascular	21	28	8	31	36	24	35	28	51	49	69	70	64
immune	3	2	2	1	1	3	4	2	5	6	7	9	10
mental	0	0	0	0	3	2	4	1	14	8	17	18	14
respiratory	27	50	23	52	44	32	48	35	78	90	98	112	105

NO_2_ – nitrogen dioxide; PM_2.5_ – particulate matter 2.5.

### Regional differences in research on the health impact of air pollution

**Figure 4.**
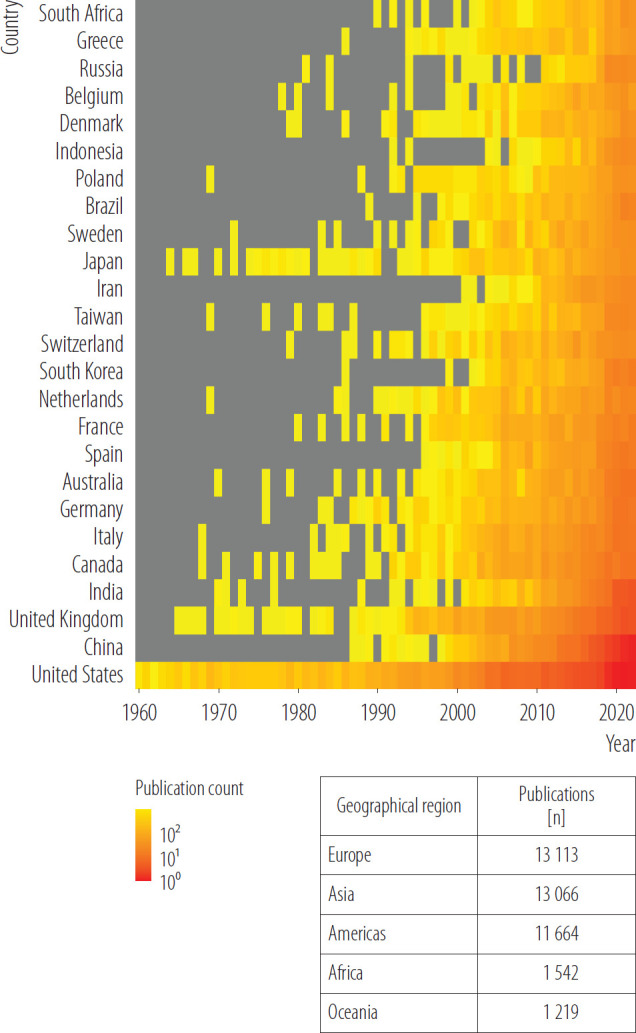
Country of origin of authors who published papers on health effects of air pollution published in 1960–2022 – a heating map, review conducted in 2024

In total, in 1960–2022 authors from 165 different countries published at least one paper on the health effects of air pollution (Figure 4). Most of the authors affiliations of papers on the health effects of air pollution were from the USA (N = 8790 authors affiliations). Since 2012, an increasing number of papers published by authors from China has been observed in English language databases. Moreover, since 1993, a constant increase in the number of papers published by authors from the United Kingdom was observed. Among all the authors who published papers on the health effects of air pollution, there was a very limited number of papers on the health effects of air pollution published by the authors affiliations from Africa (N = 1542 authors affiliations listed in the papers) or South America (N = 1042 authors affiliations listed in the papers). Details are presented in Figure 4.

### Health impact of air pollution in the scientific literature

This study is an example of the use of novel scientific database OpenAlex and digital research tools based on data mining and machine learning for bibliometric analysis. This is the first bibliometric analysis of papers on the health impact of air pollution carried out using the digital dataset of scientific papers (OpenAlex) [22–25] and text analysis data mining methods with a global reach and a long-time horizon. In this study annual numbers of papers on major air pollutions according to the WHO were presented. Moreover, particular emphasis was placed on test and language analyses that allowed for characteristics of co-occurrence of diseases and air pollutants in scientific papers as well as geographical location of author's main affiliation.

Findings from this study revealed the most common health topics in air pollution research as well as regional differences in the number of air pollution research. Respiratory diseases and cardiovascular diseases were the most common health effects of air pollution exposure analyzed in scientific papers, with a limited number of papers on mental health consequences of exposure to air pollution or the immunological response to air pollution exposure. This study also showed that most of the papers on air pollution and health were published by authors from the USA and China. Out of 193 United Nations sovereign states, authors from 165 countries published at least one paper on the health effects of air pollution.

In this bibliometric analysis, a total of 41 525 papers on the health impact of air pollution published in 1960–2022 were identified. The number of papers on the health impact of air pollution is relatively small compared to other public health issues, like smoking or diet [16,28]. Between 1960 and 1990 there were only a few papers published per year, and the increase in the annual number of scientific papers after 1990, with a significant peak after 2011 (>1000 papers published per year). This time trend corresponds to the increase in climate awareness and research interest in the issue of the impact of air pollution on anthropogenic climate change – in the early 1990s [29]. The increase in the number of published papers coincides with the publication of the first report of The Intergovernmental Panel on Climate Change (IPCC) in 1990 [30]. Another dynamic increase in the number of published papers on the health impact of air pollution occurred with the signing of the Paris Agreement on climate change in December 2015 [31]. In recent years, with subsequent IPCC reports (1995, 2001, 2007, 2014, 2021) and the tightening cooperation of the World Health Organization and World Meteorological Organization and their common initiative launched in 2018 – Collaboration Framework on Climate, Environment, and Health [32]. The presence of air pollution in the agenda of international organizations and public debate related to environmental health may have a significant impact on the research interests of scientists in the field of air pollution and its health consequences. There is a bidirectional relationship between climate change and air pollution – as some common outdoor air pollutants are also greenhouse gases and short-lived climate pollutants, so the current public debate on climate change may also contribute to the further increase in the number of papers on environmental health, including health impact of air pollution [33]. It should be noted that some increase in the number of papers on the health effects of air pollution exposure could be attributed to the increasing number of scientific journals and the growing number of researchers and scientists who are publishing more papers as well as scientific journals focused on environmental health. This bibliometric analysis revealed that particulate matter (especially PM_2.5_) is the most common air pollutant analyzed in the research on the health impact of air pollution, which is consistent with the results of narrowed systematic reviews [7]. Particulate matter 2.5 is characterized by high biological permeability and activity, and also the possibility of combining and carrying other compounds and chemical substances on particles that contribute to the harmful effect of PM_2.5_ on human health [34]. As Dominski et al. [27] stated, most systematic reviews, published between 2004–2020 look at the relationship between the effects of PM_2.5_ in cardiovascular diseases.

In the 20th century, air pollution research was focused on emissions caused by coal combustion [35] which reflects current topics in the economy and industry. This bibliometric analysis showed that research studies on gaseous pollutants such as CO, SO_2_, and O_3_ are less prevalent than studies on particulate matter. This is in line with other systematic reviews [7]. Than can result from the fact that studies on gaseous pollutants are relatively simple compared to PM indicators, and once the mechanisms for target organs are well-established [36]. Particulate matter presents multiple complexities because of its variation in particle size, chemical constituents, and target organs, and provides multi-disciplinary research opportunities for academic research communities, that may also reflect the number of papers on PM_2.5_ and PM_10_ published annually in scientific journals.

In recent years, many countries have implemented policies limiting emissions of air pollutants and are successfully striving to reduce local emissions, for example by introducing regulations regarding heating installations, production processes, etc. [15,16,37]. The problem of emission of air pollution remains still relevant. With climate change, the nature of individual exposure and the share of pollutants may increase again, e.g., due to fires caused by environmental transformation and an increase in the number of extreme meteorological phenomena [38]. Such episodes may lead to the emission of PM or other gases whose biological impact, resulting from the emission source, may present an additional or different spectrum of health burdens [39]. Increased urbanization observed in past decades raised questions on the accumulation and storage of atmospheric pollutants above the megacities and air pollution forecasting models' efficiency [40]. However, based on the example of Athens, Varotsos et al. showed that extensive photochemistry enhancement observed between 20th and 21st centuries seems not to have affected the long memory of surface ozone concentration [41]. Tendency to improve air pollution predictability is a goal for the scientific institutions and the impact of different factors on air pollution predictability should be analyzed. Some air pollutants decrease all over the world due to technological changes (e.g., CO or SO_2_), which may result in lower research interest in these gaseous pollutants.

Most of the papers on the health impact of air pollution are focused on respiratory health. The impact of air pollution on the lungs and respiratory tract is well-described in the literature [42,43]. Air pollution, especially fine particles enters the body through the lungs and can disperse to the bloodstream and be distributed throughout the human body [44]. Moreover, in vitro studies confirmed that air pollution exposure evoked oxidative stress, actual inflammation, and modulation of the immune system [45]. This biological mechanism can evoke different health effects in the human body. A significant number of papers on the health effects of air pollution are also focused on cardiovascular health and the role of air pollution in cancer development. In line with the current medical trends, findings from this analysis revealed an increase in the number of research on air pollution and mental health in the past 2 decades.

Findings from this study also revealed that most of the papers on the health impact of air pollution are focused on one group of air pollutants, mostly on PM_2.5_ and PM_10_. Various air pollutants persist in the atmosphere for many days after emission (usually 7–21) [46] and their co-occurrence in the atmosphere generates the need for research on the co-occurrence of various air pollutants and their synergistic action in the development of diseases. The high number of papers on particulate matter and its health effects may also result from the fact that the impact of PM on human health is complex and analysis of these mechanisms requires numerous investigations. In this study, a long period of observation was conducted to precisely analyze the number of papers on the health effects of air pollution over a long time period.

In this study, the country of origin listed in the affiliation of authors of papers on the health impact of air pollution was identified. Findings from this study showed that >40% of authors who published on the health impact of air pollution worked in the USA and China. Out of 193 United Nations sovereign states, there were no papers from 28 countries. For 62 years only 1542 authors affiliations from Africa and 1042 authors affiliations from South America were recorded on the health impact of air pollution, compared to over 8700 authors affiliations from the USA (single country vs. each continent). This study revealed geographic disparities in the papers on the health impact of air pollution published by scientists from lowand middle-income countries [47].

This study has numerous limitations. This bibliometric analysis is based on the abstract screening with the OpenAlex dataset and analytical tools. Full-texts of the papers were not analyzed due to limited access and copyright issues. OpenAlex is a new digital tool used, e.g., for bibliometric analysis, so further development of the codes and algorithms may contribute to more precise text analysis. Papers published after 1960 were included. In this study, co-occurrence between 6 major air pollutants of public health concern and the most common groups of diseases were analyzed. The list of air pollutants was limited to those listed by WHO [1,3,4], as major air pollutants of public health concern. In this study, PM_2.5_ and PM_10_ were the only particulate matter included in the analysis, and other particulate matter metrics (including those historical ones like “black smoke”) were not captured. Widespread measurement of PM_10_ started in 1990 whereas PM_2.5_ monitoring started in 2000, so findings on the absolute number of papers on particulate matter (especially those in the 20th century) are not free from limitations. In this bibliometric analysis, full-text papers and review papers were not separated. The list of countries and authors' affiliations were limited to the current list of 193 United Nations sovereign states and countries that existed in the last 60 years, e.g., Yugoslavia or the Soviet Union were not included in the analysis. The methods used in this study do not allow precise identification of the countries where the health effects of air pollution exposure were measured. For example, authors affiliated with, e.g., Canada analyzed air pollution in China. Moreover, if there were >2 authors from 1 country, each was calculated as a separate author from the country and added to the global number of papers from this country. In the case of multi-authored papers, this approach may lead to an overestimation of the number of papers published by the authors assigned to particular countries. Each publication was counted as a separate event so the same author can be counted multiple times if publish numerous papers on the health effects of air pollution. Due to technical reasons, the authors were not able to distinguish the epidemiological studies from the health impact assessment (HIA). The OpenAlex tools does not allow distinguishing papers by type of research. As this is the first bibliometric analysis on air pollution using OpenAlex, there is a further need for the bibliometric analyses focused on more targeted topics related to environmental research.

## CONCLUSIONS

This study confirmed that novel scientific dataset OpenAlex and AI-based research methods may be useful in bibliometric analysis. This bibliometric analysis provided detailed data on the research on the health effects of air pollution published in 1960–2022. Particulate matter, especially PM_2.5_ is the most common air pollutant included in research on air pollution and health, with a constantly growing number of papers published in the last 2 decades. Respiratory diseases were the most common health effects of air pollution analyzed in scientific papers, along with a growing number of papers on the cardiovascular effects of air pollution exposure and environmental risk factors for cancers. The most common co-occurrence pattern was PM_2.5_ exposure and lung diseases. Most of the papers were published by authors from the global North with a very limited number of papers on air pollution and health published by the authors from Africa and South America.
